# Supplementary material for: Integrated Assessment of Fungi Contamination and Mycotoxins Levels Across the Rice Processing Chain
Source: Toxins (Basel). 2025 Sep 18;17(9):468. doi: 10.3390/toxins17090468 (PMC12474497; doi:10.3390/toxins17090468)
Supplement: Supplementary file 1 [file toxins-17-00468-s001.zip › toxins-3826607-supplementary.pdf]

toxins

MDPI

From Farm to Fork: Identifying Fungi and Mycotoxins in Rice

Table S1. Linearity, recovery (n=4), intra-day and inter-day repeatability (n=5), limit of quantification, and limit of detection of the method used for mycotoxins quantification.

White Rice

Mycotoxin	Range linearity	R^2	Recovery (%)			Intra-day/Inter-day precision (RSDs, %)			LOD (µg/Kg)	LOQ (µg/Kg)
			5 (µg/Kg)	20 (µg/Kg)	80 (µg/Kg)	5 (µg/Kg)	20 (µg/Kg)	80 (µg/Kg)		
3-AcDON	12.5-600	0.9946	73	71	89	13/11	18/7	18/15	0.2	0.5
15-AcDON	12.5-600	0.9849	71	111	87	5/13	13/18	15/18	0.1	0.3
AFB1	2.5-160	0.9646	102	101	118	3/3	3/2	14/5	0.3	1.0
AFB2	2.5-160	0.9985	84	80	104	12/14	6/5	16/7	0.4	1.3
AFG1	2.5-160	0.9658	80	93	112	4/4	6/2	7/4	0.3	1.0
AFG2	2.5-160	0.9619	95	90	106	11/12	8/3	18/8	0.3	0.8
BEA	2.5-160	0.9846	87	84	105	7/19	12/6	13/7	0.4	1.3
CIT	2.5-120	0.9875	106	114	109	7/12	13/17	18/12	0.6	1.9
CPZ	2.5-160	0.9579	72	95	96	10/8	5/6	3/8	0.3	0.9
DON	12.5-400	0.9667	120	95	91	12/3	2/12	9/5	2.0	4.5
ENNA	2.5-120	0.9603	71	74	115	7/17	7/13	13/4	0.3	1.0
ENNA1	2.5-160	0.9865	71	94	73	7/17	9/13	16/4	0.5	1.4
ENNB	2.5-160	0.9860	95	76	71	9/17	11/19	15/4	0.4	1.3
ENNB1	2.5-160	0.9832	87	73	93	5/12	2/14	18/1	0.4	1.2
FB1	12.5-600	0.9773	90	84	71	3/10	13/8	7/4	0.1	0.4
FB2	12.5-600	0.9766	76	119	110	13/15	12/19	17/4	0.1	0.4
HT-2	2.5-80	0.9710	71	80	90	11/4	15/14	20/2	0.6	1.8
OTA	2.5-160	0.9926	112	114	116	4/13	4/6	1.3	0.6	1.9
STG	2.5-120	0.9728	84	85	85	3/3	7/2	1/1	0.3	0.9
TeA	2.5-160	0.9751	118	72	73	1/11	12/4	12/15	0.3	1.0
T-2	2.5-120	0.9657	106	119	72	3/14	16/16	15/11	0.4	1.1
ZEN	2.5-160	0.9723	108	80	70	12/14	15/18	14/19	0.3	0.9

Brown Rice

Mycotoxin	Range linearity	R^2	Recovery (%)			Intra-day/Inter-day precision (RSDs, %)			LOD (µg/Kg)	LOQ (µg/Kg)
			5 (µg/Kg)	20 (µg/Kg)	80 (µg/Kg)	5 (µg/Kg)	20 (µg/Kg)	80 (µg/Kg)		
3-AcDON	12.5-600	0.9979	106	72	88	14/6	17/17	2/19	0.3	1.0
15-AcDON	12.5-600	0.9471	97	99	89	15/13	16/8	10/7	0.3	1.0
AFB1	2.5-160	0.9872	75	94	78	19/10	16/9	15/16	0.4	1.4
AFB2	2.5-160	0.9923	98	97	85	17/18	16/20	13/12	0.6	1.7
AFG1	5.0-120	0.9518	116	96	84	6/7	6/11	18/18	0.3	1.0

AFG2	2.5-160	0.9817	71	99	79	14/11	16/17	10/19	0.4	1.1
BEA	2.5-160	0.9859	109	84	72	7/7	17/13	7/13	0.4	1.2
CIT	2.5-160	0.9629	74	85	90	19/10	16/17	10/2	0.3	0.8
CPZ	2.5-120	0.9485	79	85	110	19/18	13/12	7/9	0.4	1.1
DON	12.5-600	0.9962	81	96	82	10/4	17/15	3/16	0.2	0.6
ENNA	2.5-120	0.9923	101	84	106	7/4	17/11	8/5	0.7	2.2
ENNA1	2.5-160	0.996	83	76	75	13/13	6/18	13/7	0.8	2.4
ENNB	2.5-160	0.9924	101	80	105	11/10	18/15	7/10	0.6	1.7
ENNB1	2.5-160	0.9976	93	79	83	5/3	14/11	8/9	1.0	3.1
FB1	12.5-600	0.9229	81	82	99	18/9	11/7	18/11	0.3	0.8
FB2	1.5-400	0.9713	103	109	116	14/11	15/8	17/8	0.3	0.8
HT-2	2.5-160	0.9821	100	103	108	3/19	7/20	2/14	0.4	1.2
OTA	2.5-160	0.9938	94	98	117	15/14	10/19	14/3	0.6	1.9
STG	2.5-120	0.9091	86	114	96	10/12	17/19	16/19	0.3	0.7
TeA	2.5-160	0.9823	83	102	97	10/4	15/7.1	10/12	0.3	1.0
T-2	2.5-160	0.9543	85	98	100	12/14	17/16	9/15	0.3	0.8
ZEN	2.5-160	0.9870	104	103	91	19/14	15/11	4/6	0.4	1.2

R2: Coefficient of determination; RDS: relative standard deviation; LOD: Limit of detection; LOQ: Limit of quantification; 3-AcDON: 3-acetyl-deoxynivalenol; 15-AcDON: 15-acetyl-deoxynivalenol; AFB1: aflatoxin B1; AFB2: aflatoxin B2; AFG1: aflatoxin G1; AFG2: aflatoxin G2; BEA: beauvericin; CIT: citrinin; CPZ: cyclopiazonic acid; DON: deoxynivalenol; ENNA: enniatin A; ENNA1: enniatin A1; ENNB: enniatin B; ENNB1: enniatin B1; FB1: fumonisin B1; FB2: fumonisin B2; HT-2: HT-2 toxin; OTA: ochratoxin A; STG: sterigmatocystin; TeA: tenu-azonic acid; T-2: T-2 toxin; ZEN: zearalenone.

Table S2. UHPLC-MS/MS parameters for mycotoxins under study.

Mycotoxin	Retention time (min)	Molecular weight[a]	Precursor ion (m/z)	Product ions (m/z)	Collision energy (V)	Fragmentor voltage (V)	ESI mode
3-AcDON	6.04	338.4	339.2	175.1<br>203.0<br>**231.1**	25<br>15<br>5	105	Positive
15-AcDON	6.04	338.4	339.1	**137.0**<br>261.1<br>321.1	5	125	
AFB1	6.42	312.27	313.1	115.1<br>128.1<br>241.1<br>**285.1**	80<br>80<br>40<br>20	130	
AFB2	6.33	314.29	315.0	259.0<br>270.8<br>**287.0**	40<br>40<br>20	160	
AFG1	624	328.27	329.0	**243.0**<br>310.9	20	150	
AFG2	6.16	330.29	331.0	**189.0**<br>217.0	40	150	

				244.9			
BEA	8.59	783.9	784.3	**134.1**<br>244.2	70<br>20	200	
CIT	6.63	250.25	251.1	205.0<br>**233.0**	20<br>10	100	
CPZ	7.66	336.38	337.1	182.1<br>**196.1**	20	125	
DON	3.61	296.31	297.2	**174.9**<br>203.1<br>231.1	25<br>15<br>15	65	
ENNA	9.09	681.9	699.4	**100.2**<br>210.2<br>682.4	80<br>40<br>10	65	
ENNA1	8.85	667.9	685.4	**100.1**<br>210.0	80<br>40	60	
ENNB	8.47	639.8	657.3	**196.1**<br>214.1	40	75	
ENNB1	8.65	653.8	671.4	**196.2**<br>210.2	40	65	
FB1	6.85	721.8	722.4	**334.1**<br>352.1<br>704.0	40<br>30<br>40	175<br>175<br>106	
FB2	7.54	705.8	706.3	318.3<br>**336.3**	40	65	
HT-2	6.87	424.5	442.2	215.0<br>**263.1**	5<br>15	105	
OTA	7.35	403.8	404.0	**239.0**<br>358.0	20<br>10	65	
OTA-d5	7.34	408.8	409.1	238.9<br>**363.0**	20<br>10	125	
STG	7.66	324.3	325.0	280.9<br>**310.0**	40<br>20	125	
$^{13}C_{18}$-STG	7.66	342.152	343.1	**297.0**<br>326.9	40<br>20	50	
T-2	7.16	466.5	484.2	**185.1**<br>215.1<br>305.1	25<br>15<br>15	65	
ZEN	7.52	318.4	319.2	157.0<br>**283.1**<br>301.1	35<br>15<br>5	85	
TeA	6.41	197.23	196.1	112.0<br>**139.1**	20	160	Negative

3-AcDON, 3-acetyl-deoxynivalenol; 15-AcDON, 15-acetyl-deoxynivalenol; AFB1, aflatoxin B1; AFB2, aflatoxin B2; AFG1, aflatoxin G1; AFG2, aflatoxin G2; BEA, beauvericin; CIT, citrinin; CPZ,

cyclopiazonic acid; DON, deoxynivalenol; ENNA, enniatin A; ENNA1, enniatin A1; ENNB, enniatin B; ENNB1, enniatin B1; FB1, fumonisin B1; FB2, fumonisin B2; HT-2, HT-2 toxin; OTA, ochratoxin A; OTA-d5, ochratoxin d5; STG, sterigmatocystin; $^{13}C_{18}$STG, $^{13}C_{18}$ sterigmatocystin; T-2, T-2 toxin; ZEN, zearalenone; TeA, tenuazonic acid. [a]: Data from PubChem. Transition in bold was used for quantification.

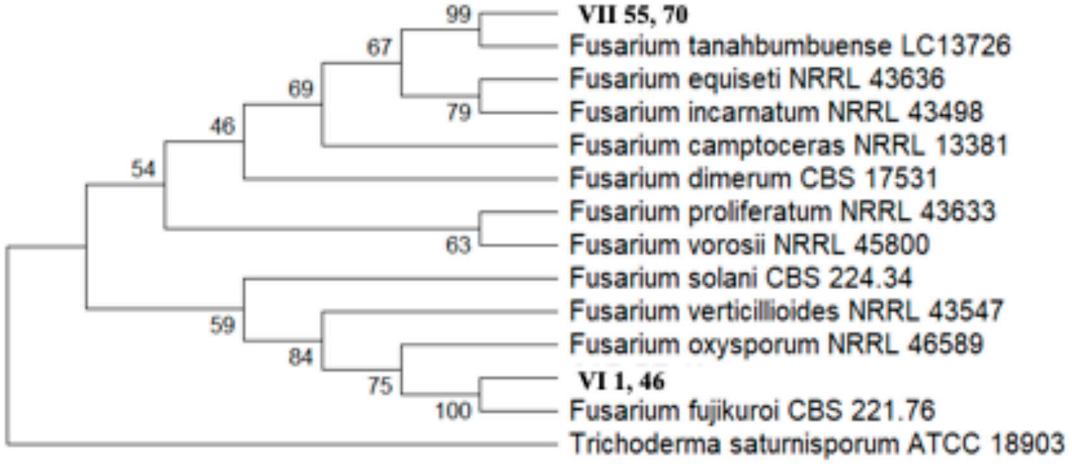

Figure S1. MEGA12- Maximum Likelihood tree of Fusarium based on aligned EF barcoding sequences.

The phylogeny was inferred using the Maximum Likelihood method and Kimura (1980) 2-parameter model [49] of nucleotide substitutions and the tree with the highest log likelihood (-4,436.05) is shown. The percentage of replicate trees in which the associated taxa clustered together (500 replicates) is shown next to the branches [50]. The initial tree for the heuristic search was selected by choosing the tree with the superior log-likelihood between a Neighbor-Joining (NJ) tree and a Maximum Parsimony (MP) tree. The NJ tree was generated using a matrix of pairwise distances computed using the Kimura (1980) 2-parameter model [49]. The MP tree had the shortest length among 10 MP tree searches, each performed with a randomly generated starting tree. The evolutionary rate differences among sites were modeled using a discrete Gamma distribution across 2 categories (+G, parameter = 0.5241). The analytical procedure encompassed 14 nucleotide sequences with 659 positions in the final dataset. Evolutionary analyses were conducted in MEGA12 utilizing up to 4 parallel computing threads. The tree is rooted using the *Trichoderma saturnisporum*. The references in bold are the fungi isolated in this study.

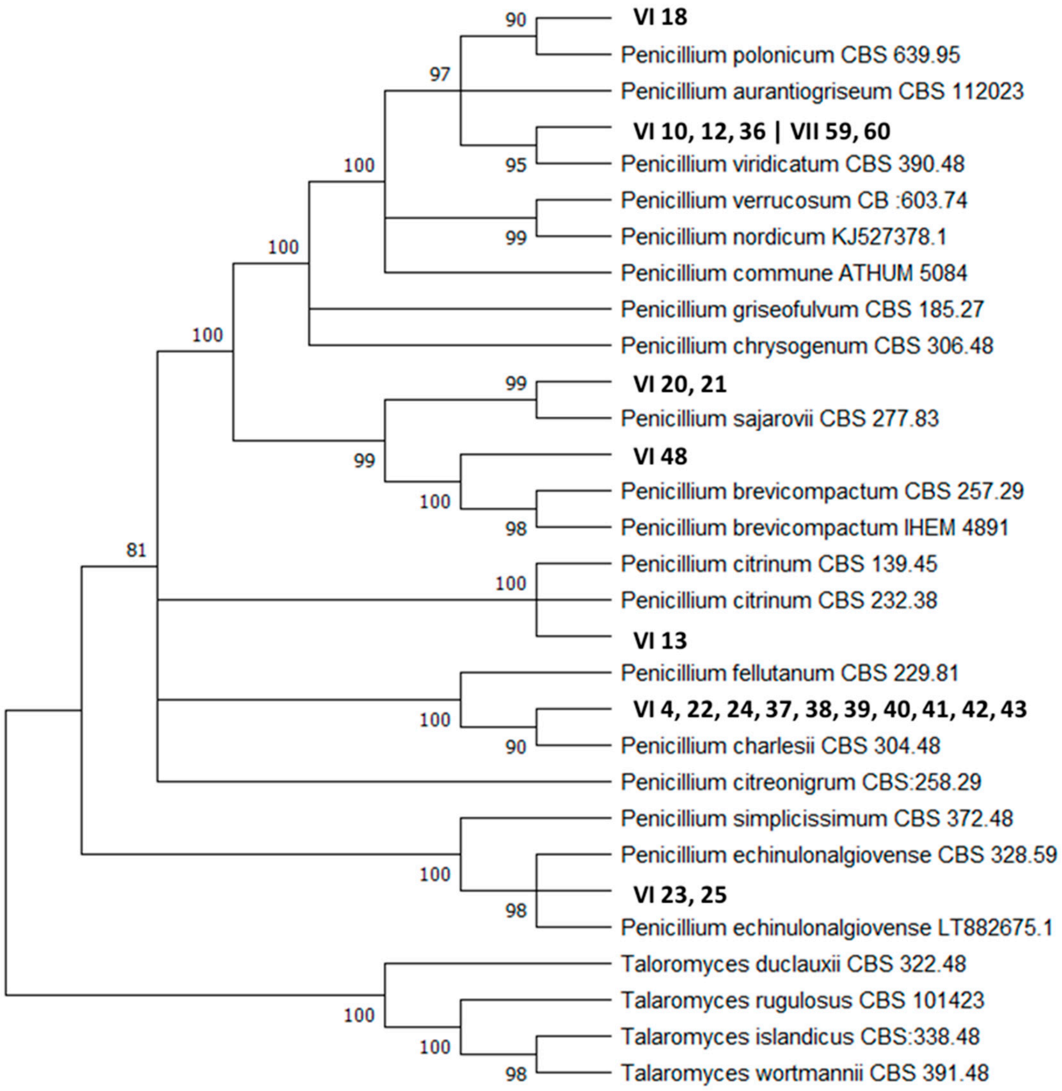

Figure S2. MEGA12- Maximum Likelihood tree of *Penicillium* based on aligned RPB2 barcoding sequences.

The phylogeny was inferred using the Maximum Likelihood method and Kimura (1980) 2-parameter model [49] of nucleotide substitutions and the tree with the highest log likelihood (-6,640.01) is shown. Branches corresponding to partitions reproduced in less than 80% of replicate trees are collapsed. The percentage of replicate trees in which the associated taxa clustered together (500 replicates) is shown next to the branches [50]. The initial tree for the heuristic search was selected by choosing the tree with the superior log-likelihood between a Neighbor-Joining (NJ) tree and a Maximum Parsimony (MP) tree. The NJ tree was generated using a matrix of pairwise distances computed using the Kimura (1980) 2-parameter model [49]. The MP tree had the shortest length among 10 MP tree searches, each performed with a randomly generated starting tree. The evolutionary rate differences among sites were modeled using a discrete Gamma distribution across 2 categories (+G, parameter = 0.2610). The analytical procedure encompassed 30 nucleotide sequences with 912 positions in the final dataset. Evolutionary analyses were conducted in MEGA12 utilizing up to 4 parallel computing threads. The tree is rooted using the *Talaromyces* species. The references in bold are the fungi isolated in this study.

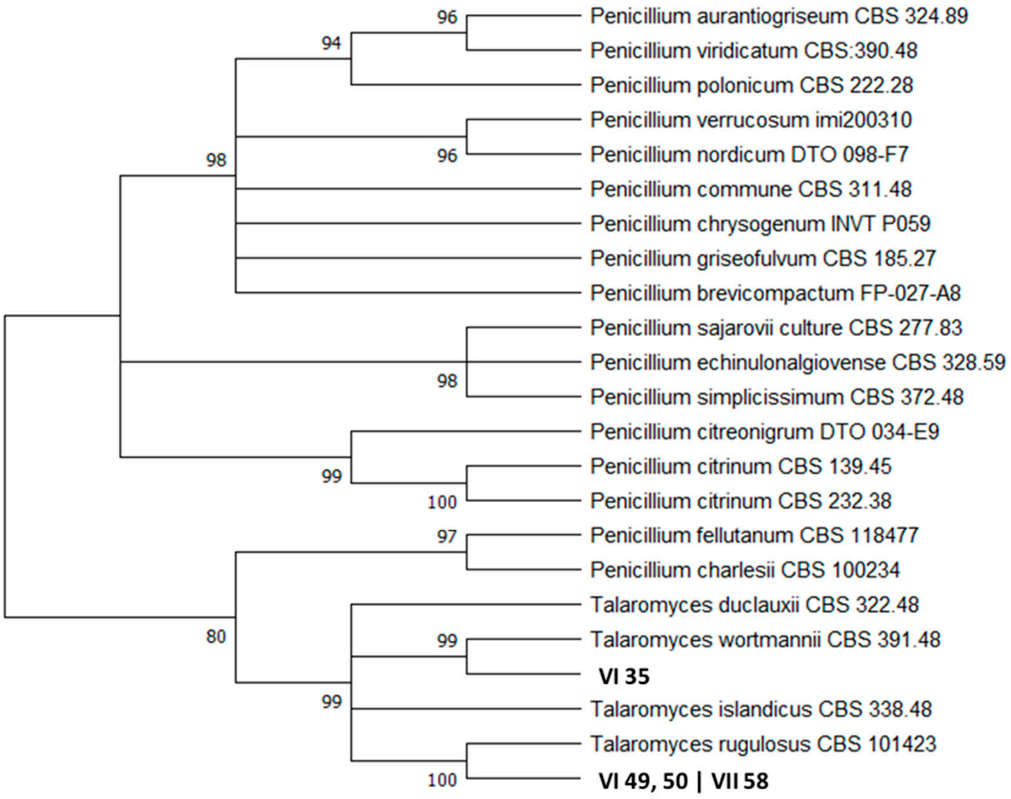

Figure S3. MEGA12- Maximum Likelihood tree of *Talaromyces* based on aligned CaM barcoding sequences.

The phylogeny was inferred using the Maximum Likelihood method and Kimura (1980) 2-parameter model [49] of nucleotide substitutions and the tree with the highest log likelihood (-2,775.06) is shown. Branches corresponding to partitions reproduced in less than 80% of replicate trees are collapsed. The percentage of replicate trees in which the associated taxa clustered together (500 replicates) is shown next to the branches [50]. The initial tree for the heuristic search was selected by choosing the tree with the superior log-likelihood between a Neighbor-Joining (NJ) tree and a Maximum Parsimony (MP) tree. The NJ tree was generated using a matrix of pairwise distances computed using the p-distance. The MP tree had the shortest length among 10 MP tree searches, each performed with a randomly generated starting tree. The evolutionary rate differences among sites were modeled using a discrete Gamma distribution across 2 categories (+G, parameter = 1.2821), with 31.57% of sites deemed evolutionarily invariant (+I). The analytical procedure encompassed 23 nucleotide sequences with 415 positions in the final dataset. Evolutionary analyses were conducted in MEGA12 utilizing up to 4 parallel computing threads. The tree is rooted using the *Penicillium* species. The references in bold are the fungi isolated in this study.

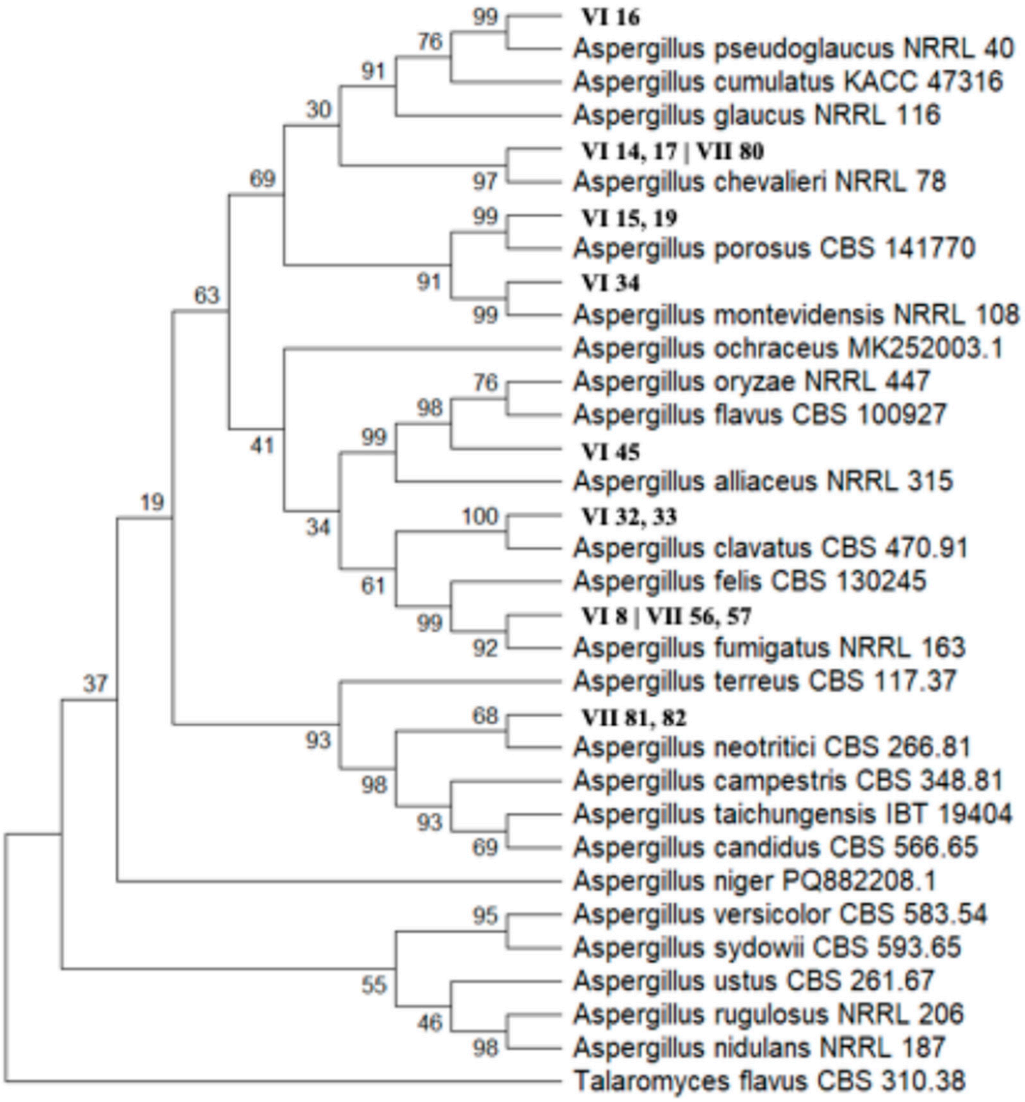

Figure S4. MEGA12- Maximum Likelihood tree of *Aspergillus* based on aligned CaM barcoding sequences.

The phylogeny was inferred using the Maximum Likelihood method and Kimura (1980) 2-parameter model [49] of nucleotide substitutions and the tree with the highest log likelihood (-3,817.13) is shown. The percentage of replicate trees in which the associated taxa clustered together (500 replicates) is shown next to the branches [50]. The initial tree for the heuristic search was selected by choosing the tree with the superior log-likelihood between a Neighbor-Joining (NJ) tree and a Maximum Parsimony (MP) tree. The NJ tree was generated using a matrix of pairwise distances computed using the Kimura (1980) 2-parameter model [49]. The MP tree had the shortest length among 10 MP tree searches, each performed with a randomly generated starting tree. The evolutionary rate differences among sites were modeled using a discrete Gamma distribution across 2 categories (+G, parameter = 1.2127), with 32.64% of sites deemed evolutionarily invariant (+I). The analytical procedure encompassed 33 nucleotide sequences with 432 positions in the final dataset. Evolutionary analyses were conducted in MEGA12 utilizing up to 4 parallel computing threads. The tree is rooted using the *Talaromyces flavus*. The references in bold are the fungi isolated in this study.

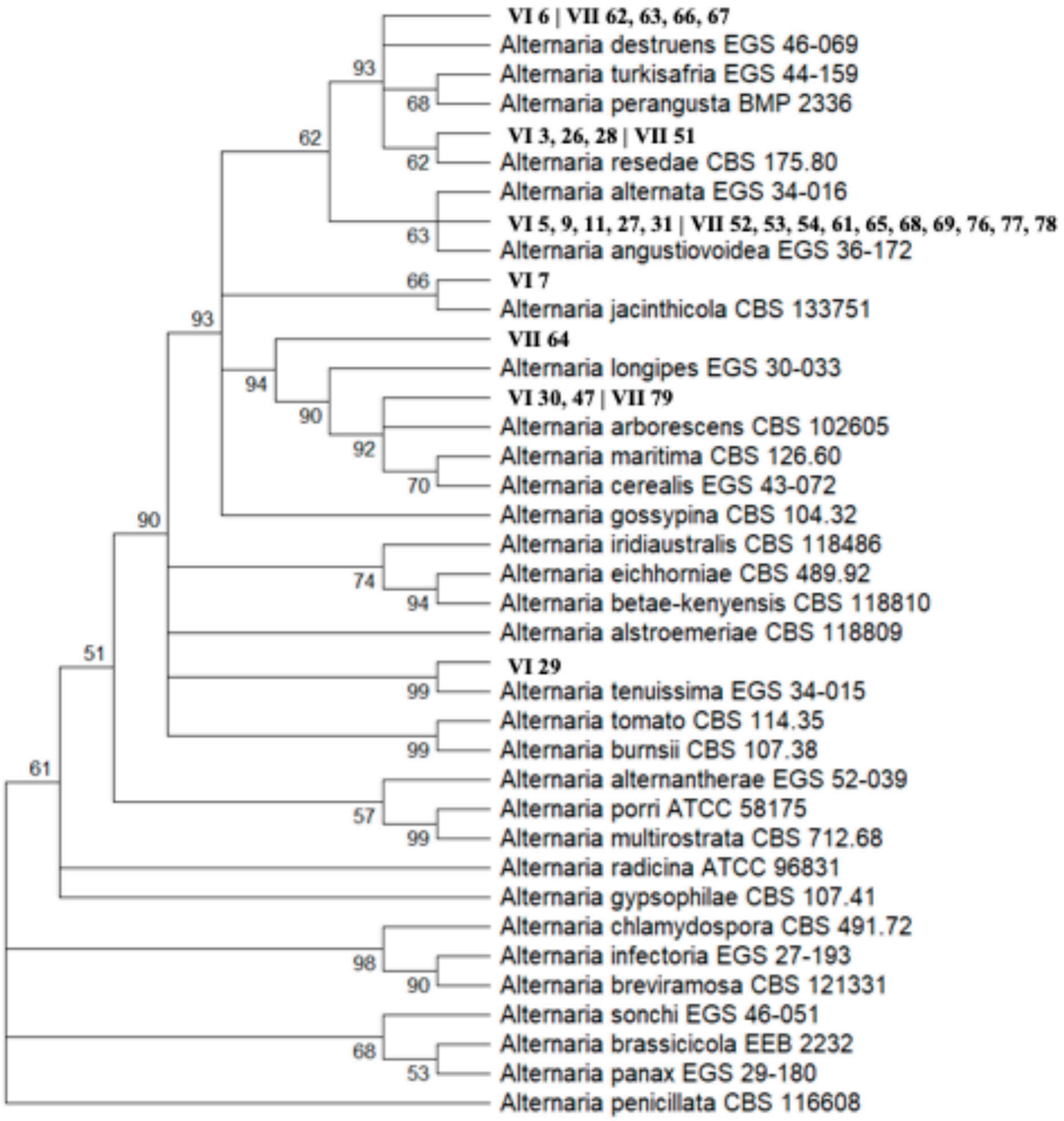

Figure S5. MEGA12- Maximum Likelihood tree of *Alternaria* based on aligned ATPase barcoding sequences.

The phylogeny was inferred using the Maximum Likelihood method and General Time Reversible model [49] of nucleotide substitutions and the tree with the highest log likelihood (-4,051.82) is shown. Branches corresponding to partitions reproduced in less than 70% of replicate trees are collapsed. The percentage of replicate trees in which the associated taxa clustered together (1,000 replicates) is shown next to the branches [50]. The initial tree for the heuristic search was selected by choosing the tree with the superior log-likelihood between a Neighbor-Joining (NJ) tree and a Maximum Parsimony (MP) tree. The NJ tree was generated using a matrix of pairwise distances computed using the General Time Reversible model [49]. The MP tree had the shortest length among 10 MP tree searches, each performed with a randomly generated starting tree. The evolutionary rate differences among sites were modeled using a discrete Gamma distribution across 2 categories (+G, parameter = 1.7831), with 59.45% of sites deemed evolutionarily invariant (+I). The analytical procedure encompassed 38 nucleotide sequences with 986 positions in the

final dataset. Evolutionary analyses were conducted in MEGA12 utilizing up to 4 parallel computing threads. The tree is rooted using the *Alternaria penicillata*. The references in bold are the fungi isolated in this study.
